# Kif4A mediate the accumulation and reeducation of THP-1 derived macrophages via regulation of CCL2-CCR2 expression in crosstalking with OSCC

**DOI:** 10.1038/s41598-017-02261-7

**Published:** 2017-05-22

**Authors:** Yun Zhang, Shaohua Liu, Daiwei Qu, Ketao Wang, Lin Zhang, Xuanxuan Jing, Chen Li, Fengcai Wei, Xun Qu

**Affiliations:** 1 Institute of Stomatology, Qilu Hospital of Shandong University, Jinan, 250012 Shandong P.R. China; 2 Department of Gynaecology and Obstetrics, Qilu Hospital of Shandong University, Jinan, 250012 Shandong P.R. China; 3 Institute of Basic Medical Sciences, Qilu Hospital of Shandong University, Jinan, 250012 Shandong P.R. China

## Abstract

Crosstalk between tumor infiltrating macrophages and tumor cells is thought to play an indispensable role in oral squamous cell carcinomas (OSCC) by induction and maintenance of tolerance microenvironment. High infiltration of M2 macrophages and increasing expression of Kinesin family member 4A (Kif4A) in primary OSCC have been proved to correlate with greater tumoral size and poor clinical outcome. However, linkage between Kif4A and infiltrating macrophages in tumorigenesis and progression remains unclear. In the present study, we show that, the interaction between THP-1derived macrophage and OSCC cell line Cal-27 may up-regulate the Kif4A expression in both of them. Additionally, elevated soluble CCL2 in medium and more expression of CCR2 on macrophage were observed during the crosstalk. SiRNA of Kif4A and neutralizing antibody of CCL2 were utilized to identify that; increasing Kif4A can promote the recruitment of macrophages towards Cal-27 and educate them to M2 polarized macrophages via regulating CCL2/CCR2. In combination, the results of the present study may provide interesting clues to understanding the Kif4A-CCL2/CCR2-macrophage axis as a novel therapeutic target to improve the clinical outcome of OSCC.

## Introduction

Oral squamous cell carcinoma (OSCC), being a major part of head and neck squamous cell carcinoma (HNSCC), accounts for almost 90% of all oral malignancies^1, 2^. Despite previous researches have made tremendous efforts to improve in the field of surgery, radiotherapy and chemotherapy, the mortality rates failed to descend substantially. Growing study suggests that tumor-immunological parameters in the prognostic classification and treatment of OSCC should be considerate sufficiently^3, 4^. As a kind of high-plastic immunocyte, macrophages constitute a major component of leukocytes in almost all types of malignancy including OSCC. It responds to the stimulator in varied tumor microenvironments and be polarized with the release of a far-ranging of cytokines, chemokines and enzymes that mediate cancer cells invasion into surrounding normal tissues, and metastasis to local or distant sites^5^. Accumulating evidences indicate a close association of alternative polarization of macrophages (M2) with an increased dedifferentiation and poor cancer outcome, which can already be detected in the initial biopsies in early stage of OSCC^6, 7^. Thus, the mechanisms of macrophages recruitment at the tumor sites and interaction with cancer cells are gaining increasing attention in the OSCC progression. But the process how OSCC cells attract macrophages and polarize them with an immune tolerance feature remains incompletely understood.

A possible candidate for this role is represented by chemokine C-C motif ligand (CCL2), which has been found in many kinds of cancer tissue samples and function as a crucial mediator in promoting tumorigenesis and metastasis^8, 9^. Besides its capacity to inducing the recruitment of monocytes/macrophages via binding to the receptor CCR2, our previous study and many others’ have proved that elevated CCL2 contributes to the establishment of immunotolerance microenvironment via educating monocytes/macrophages to possess immunosuppressive function and stimulating T lymphocytes polarized towards T_H_2 cells^10, 11^. The versatile roles of CCL2 in both promotion of tumor progression/metastasis and education of immunocyte make the CCL2-signaling a dramatic therapeutic target for tumor treatment. Although there is a large body of evidence supporting CCL2 plays a crucial and positive regulatory role in tumorigenesis, the mechanism of their regulation in OSCC has not been fully investigated.

Kinesin superfamily (Kif) member Kif4A, is a microtubule-based motor protein that fulfill its essential role in intracellular anterograde transport and cell division^12^. It has been reported that Kif4A has close association with activation of immunocyte^13^, and our former microarray study has found a decreased expression of CCL2 and CCR2 in mouse’s macrophage cell line when Kif4A is silencing (unpublished). It is absent or present at very low level in most adult normal tissues, however, cDNA microarray study and histochemical analysis reveal that there is an over-expression of Kif4A mRNA in human cervical cancer and it might be a prognostic biomarker and a possible therapeutic target for lung cancer^14, 15^. Notably, there is also an elevated expression of Kif4A in OSCC and it was speculated to be a key regulator for tumoral progression of human oral cancer^16^.

Owing to above observations, we postulate that there would be a possible link between aberrant change of Kif4A and induction of recruited macrophages which contribute to the generation of immunotolerant tumor microenvironment. However, to the best of our knowledge, there are no definitive experiments showing that increasing Kif4A has an effort on educating immunocyte, especially macrophage, in OSCC. Therefore, the aim of resent study is to assess whether Kif4A might recruit macrophage and modulate it towards an immune tolerance feature by CCL2-signaling in OSCC.

## Results

### Both Kif4A and CCR2 are increased in THP-1 derived macrophages via co-culturing with OSCC

**Figure 1:**
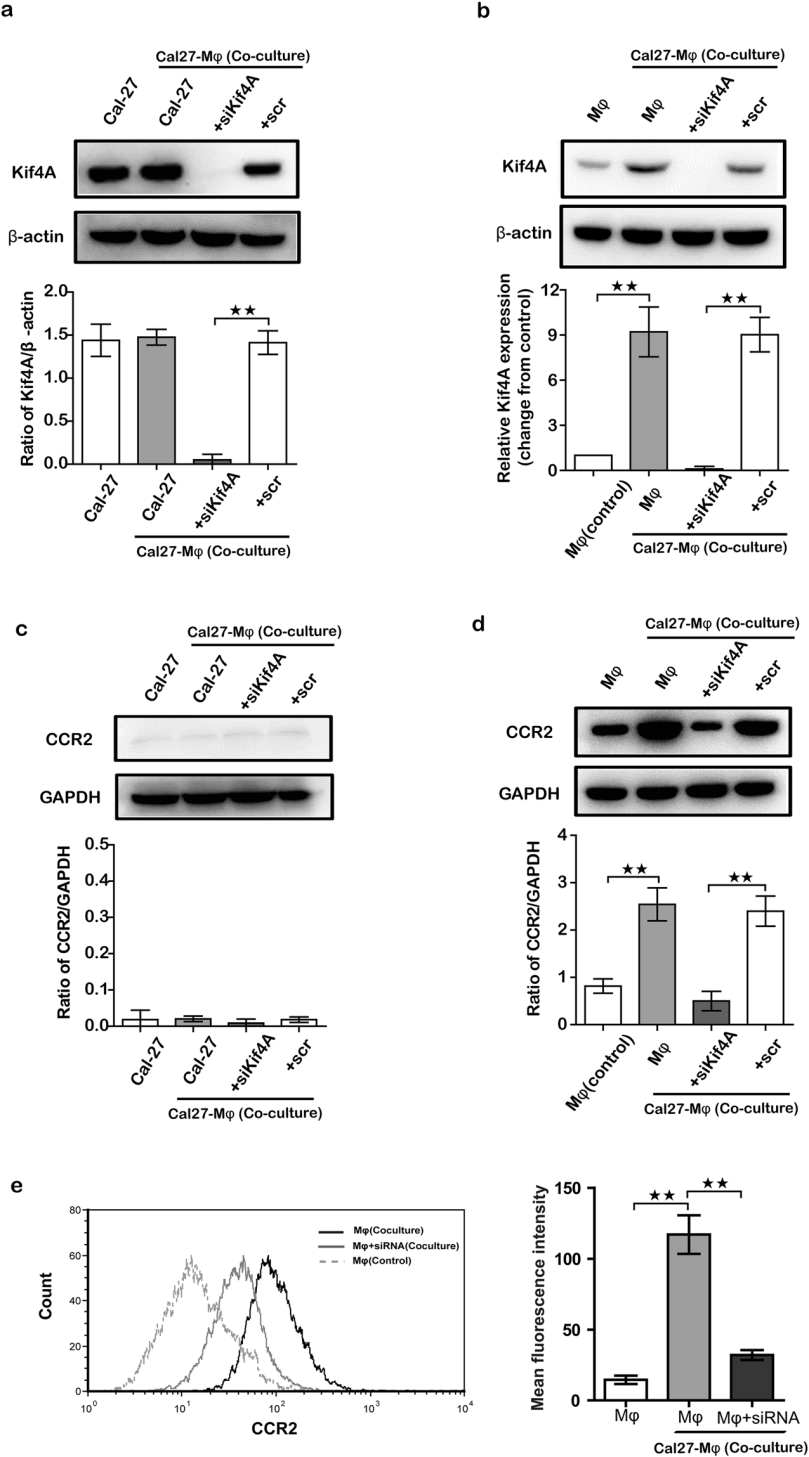
Both Kif4A and CCR2 are increased in THP-1 derived macrophages via co-culturing with OSCC. The OSCC cells line Cal-27 and THP-1-derived macrophages were pre-treated by scramble (scr) or silenced Kif4A (siKif4A) as described in the Methods for 24 h and then co-culture for another 24 h. Two types of cells cultured alone for 24 h without any pre-treating were used as control. Western blotting analyzed the expression of Kif4A and CCR2. (**a** and **b**) Kif4A protein level in Cal-27 or THP-1-derived macrophages from co-culture system or control groups were detected by Western blotting (n = 5). Respective densities of the bands normalized to β-actin. (**c** and **d**) CCR2 protein level in Cal-27 or THP-1-derived macrophages from co-culture system or control groups were detected by Western blotting (n = 5). Respective densities of the bands normalized to GAPDH. (**e**) Expression of CCR2 on the surface of THP-1-derived macrophages from different groups was analyzed by flow cytometry as described in the Methods. Shown are cumulative results from 5 independent experiments. Data are presented as the mean ± SEM. **p < 0.01.

To assess the expression and function of Kif4A in OSCC and mimic the crosstalk between macrophages and OSCC cells in the tumor microenvironment, we employed an *in vitro* two-chamber transwell co-culture system that allows the interaction between infiltrating macrophages and OSCC cells in the existence or absence of Kif4A silencing. Consistent with recent findings *in vivo* ^16^, Cal-27, a kind of OSCC cell line, expressed Kif4A in a high level (Fig. 1a). Then, we performed western blot analysis of Kif4A expression of Cal-27 and monocyte cell lines, THP-1-derived macrophages in co-culture system after 24 hours culturing. As shown in Fig. 1a, there was no obviously change of Kif4A that can be detected in Cal-27 between co-culture system and monocultures group. On the contrary, compared with the monoculture group, co-culturing with Cal-27 cells significantly increased Kif4A expression in THP-1-derived macrophages (Fig. 1b). Furthermore, we detected the relationship between Kif4A and macrophage in OSCC tissue by immunohistochemistry. As the same with our observation in co-culture system, the expression of Kif4A in OSCC tumor cells was at a high level and tumor-infiltrating macrophages express Kif4A at a middle level (as shown in the black arrow). However, we cannot detect Kif4A expressing macrophage in pericarcinous tissue (see Supplementary Fig. S1).

Then, we silenced Kif4A function via liposome Kif4A-siRNA (siKif4A) and used scramble RNA (scr) as a control. As shown in Fig. 1a and b, siKif4A can blockaded the expression of Kif4A in both Cal-27 and THP-1-derived macrophages efficiently.

To study the functional role of Kif4A, we analyzed CCR2 expression of Cal-27 and THP-1-derived macrophages in co-culture system with siKif4A or scr after 24 hours culturing. Western blot analysis showed that, CCR2 of THP-1-derived macrophages was significantly up-regulated in the presence of Cal-27. However, knockdown of Kif4A expression of macrophages in co-culture system with siKif4A was able to significantly reduce the increased level of CCR2 of macrophages (Fig. 1d). Conversely, CCR2 maintained in a very low level and no different expression was detected in Cal-27 of all groups (Fig. 1c). We also evaluated expression of CCR2 on the surface of THP-1-derived macrophage by flow cytometry. Similar results as described above by western blot analysis were observed, CCR2 level on the surface of THP-1-derived macrophage was promoted when co-cultured with Cal-27, which can also be reduced when knockdown of Kif4A expression of macrophages by siKif4A (Fig. 1e). However, we failed to detect knockdown Kif4A expression of Cal27 in co-culture system have significantly effect on the expression level of CCR2 on the surface of macrophages as compare with it from co-culture system without any treatment. Our results demonstrate that Kif4A of macrophages will be significantly up-regulated during co-culturing with OSCC cells; it may also involve in specific activation of the CCR2 of macrophages.

### Blockade of Kif4A ablates the significant expression of CCL2 in co-culture system

**Figure 2:**
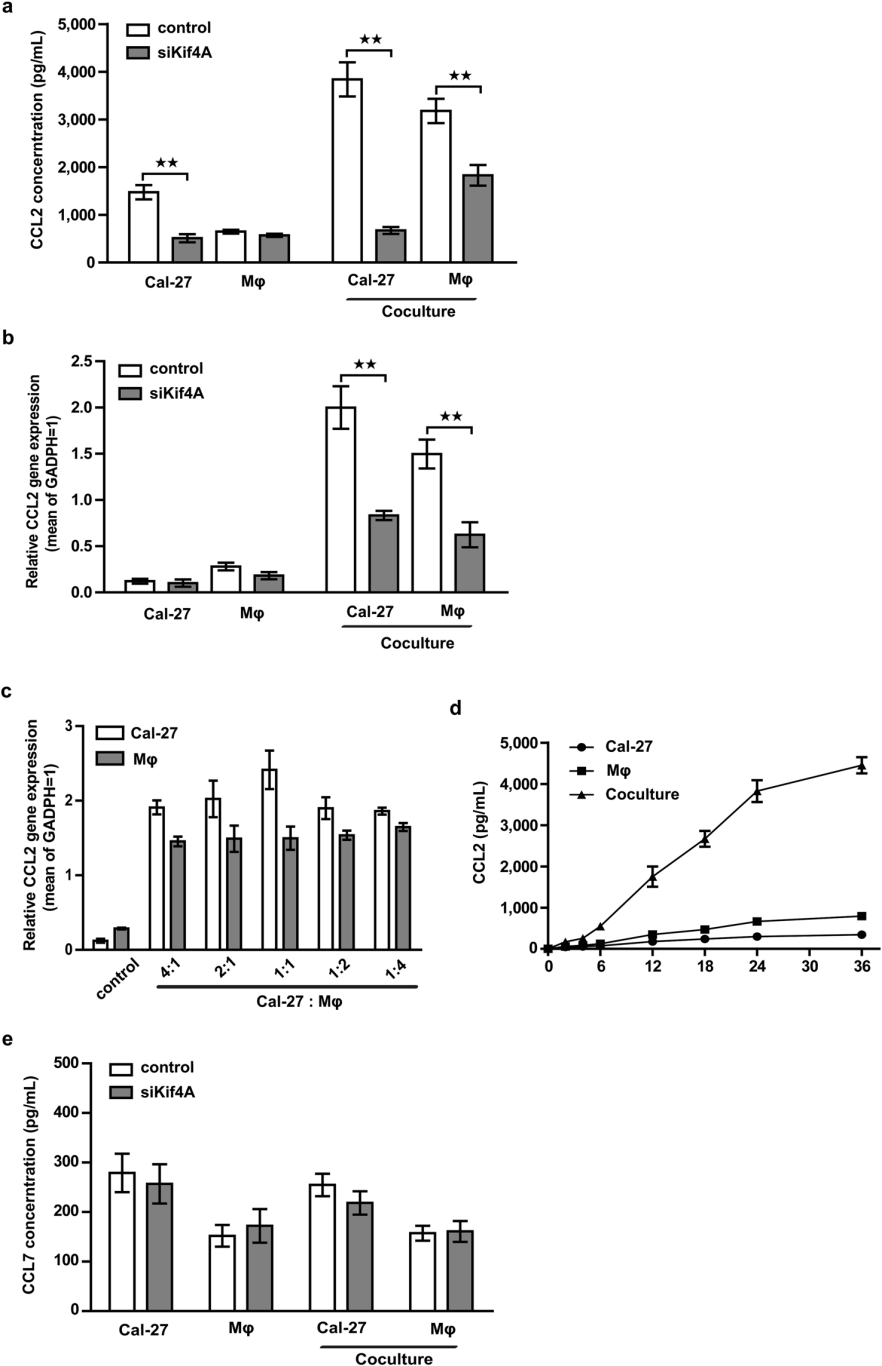
Blockade of Kif4A ablates the significant expression of CCL2. (**a**) Pre-treated by scramble (scr) and silenced Kif4A (siKif4A), Cal-27 (2 mL/well, 1 × 10^5^/mL) and THP-1-derived macrophages (1 mL/well, 2 × 10^5^/mL) were co-cultured for 24 h, the two chambers of cells were collected respectively and cultured alone in 2 × 10^5^/mL with 1 mL pre-warmed fresh medium in 48-well plate for another 24 h, and then cell-free supernatants were collected respectively. Cal-27 and THP-1-derived macrophages monocultures without any pre-treating groups were used as control. CCL2 concentrations in different groups were analyzed by ELISA. (**b**) Cells were harvested from different groups as (**a**) mentioned after co-culture for 24 h, transcription level of CCL2 were measured by RTQ-PCR as described in the Methods. (**c** and **d**) Cal-27 and THP-1-derived macrophages were co-cultured at different ratio for 24 h or at 1:1 ration for different time, CCL2 were measured by RTQ-PCR. (**e**) CCL7 concentrations in different groups as (**a**) mentioned were analyzed by ELISA. All images shown are representative of 5 independent experiments and all data are shown as the mean ± SEM. **p < 0.01.

CCL2 was thought to be the strongest ligand for CCR2, which may enhance tumors growth/metastasis *in vivo* by increasing the recruitment of macrophages and angiogenesis. More importantly, our group and others had proved that secretion factors including CCL2, vascular endothelial growth factor (VEGF), trans-forming growth factor-β (TGF-β), have been implicated as being important cell co-culture regulators. Then we investigated whether this co-culture system could affect the expression of CCL2, and whether Kif4A was also involved in this progress. After co-cultured for 24 hours, two chambers’ cells were separated and cultured alone in 2 mL fresh medium for another 24 h, supernatant was collected and CCL2 concentration was analyzed by ELISA. Results showed that secreted levels of CCL2 were remarkable elevated in both Cal-27 cells and THP-1-derived macrophage when co-cultured with each other compared with cells monoculture groups (Fig. 2a). When we treated Cal-27 cells or THP-1-derived macrophage with siKif4A, it dramatically prevented the up-regulation of CCL2, especially in the Cal-27 chambers of co-culture system (Fig. 2a). We undertook to further identify the source of co-culture-produced CCL2. To do so, we carried out quantitative real-time PCR (RTQ-PCR) and found CCL2 expression levels in both THP-1-derived macrophage and Cal-27 were increased during co-culture with each other (Fig. 2b). As expected, we found targeting Kif4A with siRNA in both THP-1-derived macrophage and Cal-27 were significantly reduced the up-regulated expression of CCL2 (Fig. 2b).

To test if this raise was associated with the ratio of macrophages to cancer cells, Cal-27 cells were co-cultured with increasing amounts of THP-1-derived macrophages. Cells were collected after 24 hours co-culture and the RTQ-PCR results showed that, transcription level of CCL2 of Cal-27 cells increased to the peak at 1:1 ratio of macrophages to cancer cells and started to decrease at 1:2 ratio. In contrast, no marked change of CCL2 transcription was found in THP-1-derived macrophages at any co-culture ratio (Fig. 2c). Next, we examined the dynamic change of secreted levels of CCL2 in different time points at 1:1 ratio of macrophages to cancer cells. We found that secretion rate of CCL2 in co-culture system became increasing after 6 hours and it was increased to about 4000 pg/mL after 24 hours, then the secretion rate slowed down (Fig. 2d). Given that CCL7 was another important ligand of CCR2, we determined whether co-culture system or Kif4A was also involved in regulation of this chemokine ligand. We observed that neither co-culture system nor Kif4A had obviously effected on the regulation of CCL7 (Fig. 2e).

### Co-culture system enhanced migration of the macrophage via Kif4A-CCL2 induction

**Figure 3:**
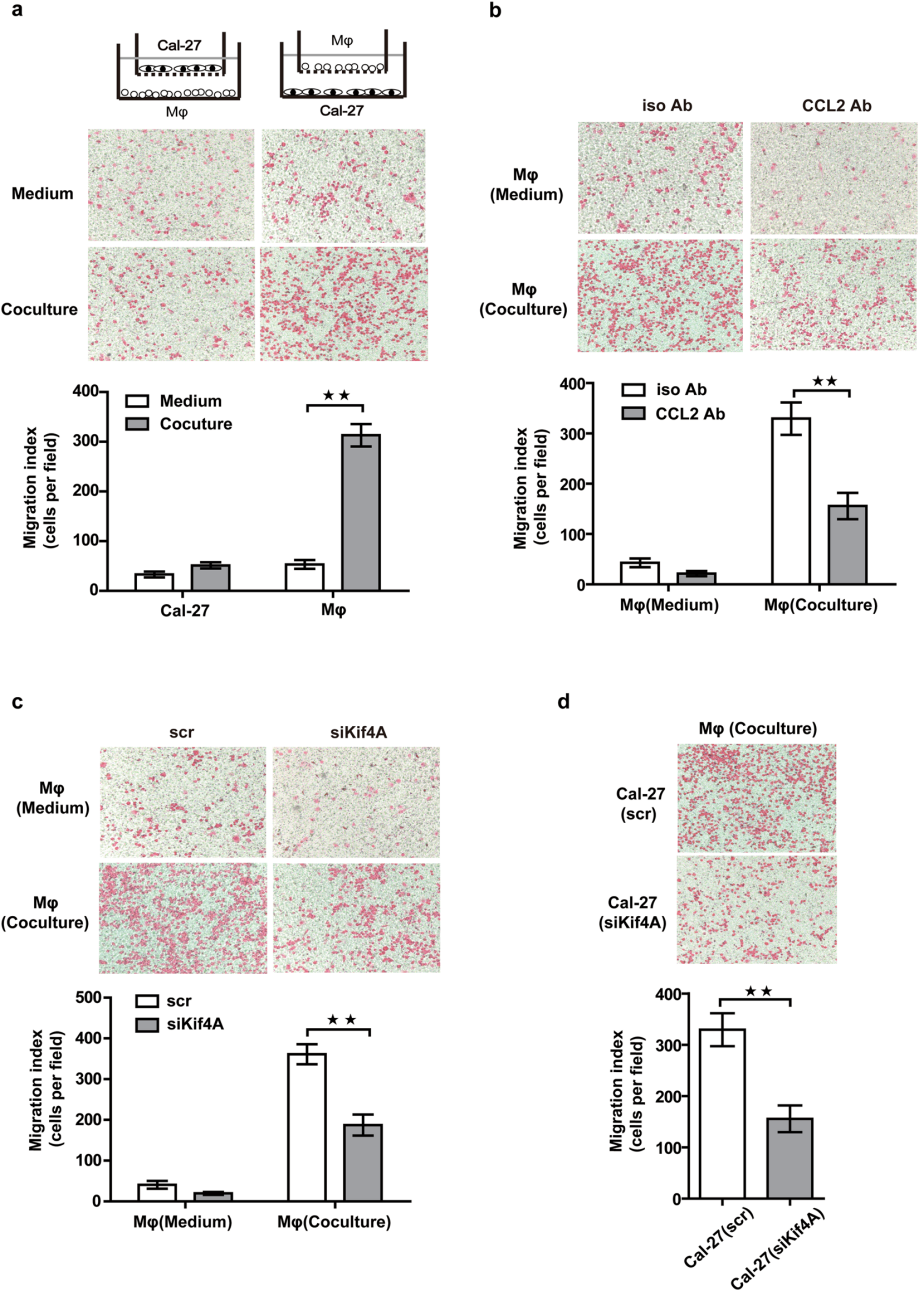
Kif4A-CCL2 promoted the recruitment of the macrophage towards Cal-27. (**a**) Migration assay of Cal-27 towards THP-1-derived macrophages and THP-1-derived macrophages towards Cal-27 was achieved as described in the Methods Medium in lower chambers without cells groups were use as control. (**b**) The migrated THP-1-derived macrophages towards Cal-27 with/without CCL2 neutralizing antibody or isotype antibodies (10 μg/mL) were counted. (**c**) Migration assay of THP-1-derived macrophages, with scr or siKif4A pre-treated, towards Cal-27 was achieved. Medium in lower chambers without cells groups were use as control. (**d**) The migrated THP-1-derived macrophages towards Cal-27, with scr or siKif4A pre-treated, were counted. Pictures were magnified at 100×, Bars in graphs (**a**–**d**) are representative of 5 independent experiments and all data are shown as the mean ± SEM. **p < 0.01.

CCR2 and its ligand CCL2 were a couple of chemotactic factors which played an important role in recruitment of macrophage^17^. In order to extend our findings with the link of Kif4A to CCR2 and CCL2, we then set out to identify recruitment effect of Kif4A on macrophages. We performed transwell assays by a two-chamber migration system, in which, THP-1-derived macrophages and Cal-27 were separated by an 8  μm pore membrane. After 24 hours, remarkable increased numbers of THP-1-derived macrophages migrated toward Cal-27 cells in the lower chamber, as compared with the lower chamber containing medium alone (Fig. 3a right). But, there was little effect on Cal-27 cells recruitment during co-culture as compared with the lower chamber containing medium alone (Fig. 3a left). We hypothesized that up-regulation of CCR2 and CCL2 is capable of stimulating chemotaxis of macrophages, since we observed robustly elevated CCL2 level in co-culture system and increased expression of CCR2 in THP-1-derived macrophages. In support of this hypothesis, we assessed the effect of CCL2 neutralization on macrophages migration in co-culture system. Anti-CCL2 neutralizing mAb (10 μg/mL) and the isotype antibody (10 μg/mL) were added to medium in the lower chamber of two-chamber migration system before the transwell assay. As shown in Fig. 3b, the addition of anti-CCL2 neutralizing mAb, but not isotype control, significantly prevented the up-regulation of macrophages migration.

Next, we investigated whether Kif4A silencing-induced decreased of secreting CCL2 in supernatant and expression of CCR2 on THP-1-derived macrophages may also effect the capable of macrophages migration. As we expected, the migration of macrophages was dramatically decreased with siKif4A treated THP-1-derived macrophages, as compared with scramble treated THP-1-derived macrophages in two-chamber migration system (Fig. 3c). Similarly, targeting Kif4A with siKif4A in Cal-27 cells of co-culture system also significantly reduced the migration of macrophages as compared with scr treated Cal-27 cells in co-culture system (Fig. 3d). In order to exclude the influence of lipofectamine on the results, we also compared cells migration without any treatment group (Fig. 3a right) with scr treated THP-1-derived macrophages group (Fig. 3c left) both in co-culture system. We observed similar cells migration ability between them, it suggested lipofectamine has no obvious influence on the cells migration in our co-culture system. Therefore, these results clearly showed that Kif4A mediated recruitment of macrophage in co-culture system via regulate secretion of CCL2 in both Cal-27 cells and THP-1-derived macrophages and expression of CCR2 on macrophages.

### Kif4A-CCL2 modulated THP-1-derived macrophages towards M2-type cytokine profile

**Figure 4:**
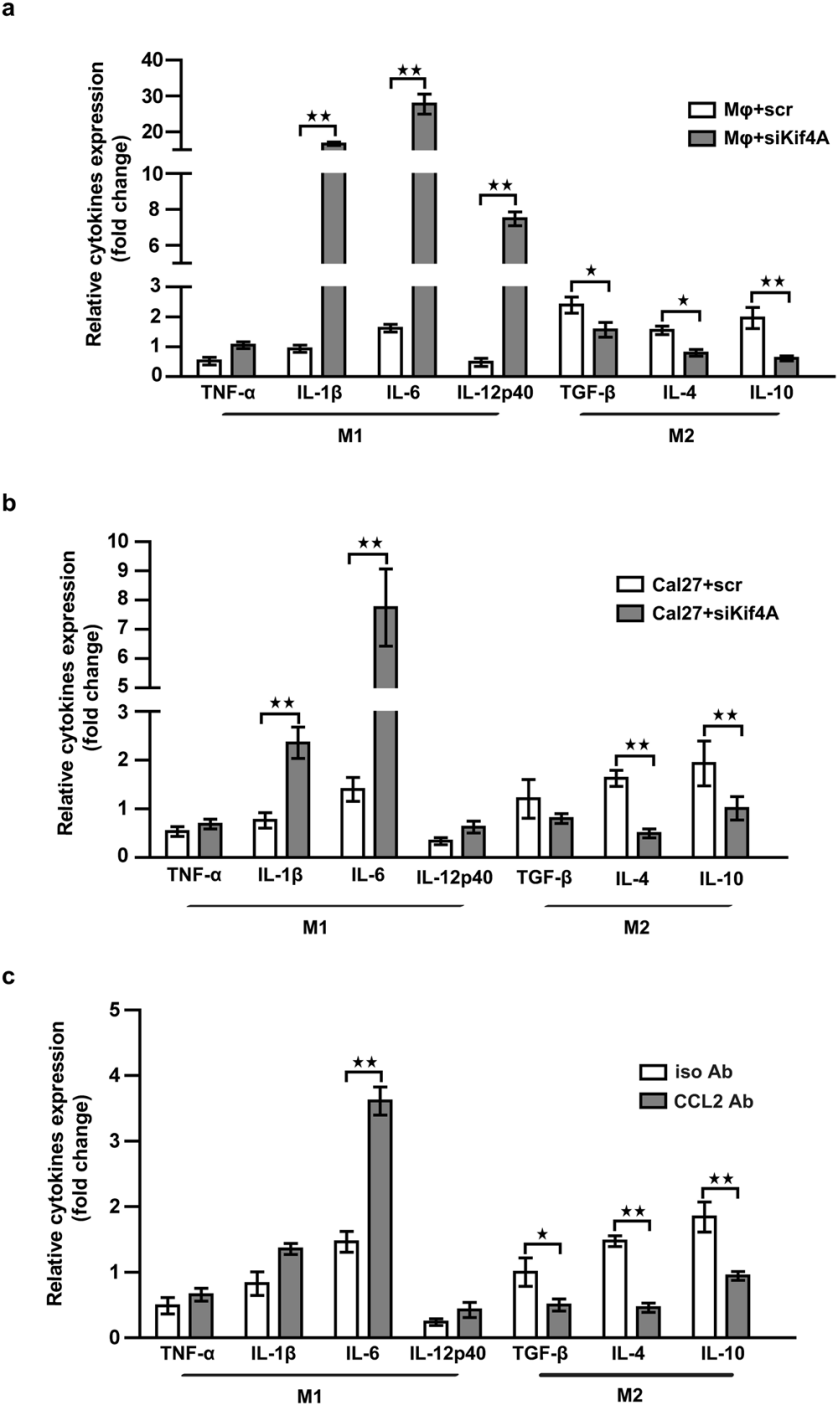
Kif4A-CCL2 modulated macrophages function. The transcription of typical pro-inflammatory or anti-inflammatory cytokines of THP-1-derived macrophages was measured by RTQ-PCR. (**a**) THP-1-derived macrophages were transfected by scr or siKif4A, and then co-cultured with Cal-27 for 24 h. (**b**)Cal-27 was transfected by scr or siKif4A, and then co-cultured with THP-1-derived macrophages for 24 h. (**c**) CCL2 neutralizing antibody or isotype antibodies (10 μg/mL) was added into supernatant of co-culture system for 24 h. Relative gene expression was normalized to the expression of GAPDH mRNA and expressed as fold change for each relative gene reaction among the samples. Each bar represents the mean ± SEM (n = 5, *p < 0.05, **p < 0.01).

Macrophages infiltrated in tumor were considered as polarized M2 macrophages population in most mouse and human tumors^5^. Our next objective was to ascertain whether co-culture system could modulate the cytokines expression of THP-1-derived macrophages towards M2-type profile. For this purpose the two-chamber co-culture system was used, where THP-1-derived macrophages were localized in the lower chamber and Cal-27 cells to the upper chamber. We tested the transcription of M1-type cytokines (TNF-α, IL-1β, IL-6 and IL-12p40) and M2-type cytokines (TGF-β, IL-4 and IL-10) by RTQ-PCR. As shown in Fig. 4a, transcription of most M1-type cytokines were enhanced but all M2-type cytokines’ were reduced in macrophages when siKif4A treated THP-1-derived macrophages in co-culture system as compared with scr treated. Moreover, when we treated Cal-27 cells in the upper chamber THP-1-derived macrophages of co-culture system with siKif4A or scr, the similar results were observed (Fig. 4b).

To explore the potential role of CCL2 in the experimental setting used in the present study, and further identify Kif4A modulated THP-1-derived macrophages towards M2-type cytokine profile via regulate the expression of CCL2, the above-mentioned experiments were performed with addition of neutralizing mAb (10  μg/mL) or the isotype antibody (10 μg/mL) to the co-culture system. As expected, when CCL2 was neutralized, transcriptions of M1-type cytokines were enhanced and M2-type cytokines’ were reduced in macrophages as compared with isotype antibody treated co-culture system (Fig. 4c).

Taken together, the cytokine profile analysis suggested, Kif4A not only take part in mediating migration of macrophages, but also involve in regulating macrophages towards M2-type. Both these processes were achieved by regulate expression of CCL2.

### Kif4A-CCL2 promoted expression of suppressive molecules of THP-1-derived macrophages via STAT3 signaling

**Figure 5:**
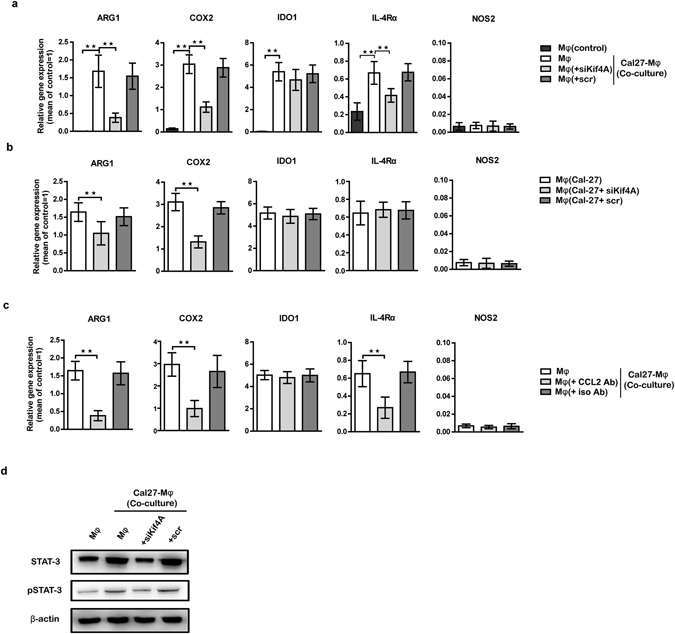
Kif4A-CCL2 promoted expression of suppressive molecules in macrophages. The transcription of typical suppressive molecules of THP-1-derived macrophages was measured by RTQ-PCR. (**a**) THP-1-derived macrophages were transfected by scr or siKif4A or not, and then co-cultured with Cal-27 for 24 h. THP-1-derived macrophages monocultures group was used as control. (**b**) Cal-27 were transfected by scr or siKif4A or not, and then co-cultured with THP-1-derived macrophages for 24 h. (**c**) CCL2 neutralizing antibody or isotype antibodies (10 μg/mL) was added into supernatant of co-culture system for 24 h. Co-culture system without any antibody was used as control. (**d**) Western blotting analyzed the expression of (*p*)STAT-3 in THP-1-derived macrophages from different groups as mentioned in (**a**). Relative gene expression was normalized to the expression of GAPDH mRNA and expressed as fold change for each relative gene reaction among the samples. All images represent five independent experiments, and all data are shown as the mean ± SEM. **p < 0.01.

THP-1 is an acute monocytic leukemia cell line, which belongs to human myelomonocytic cells. Our previous study showed that, CCL2 was involved in the induction of candidate suppressive molecules of myelomonocytic cell-derived MDSCs^10^. On the basis of this result, we hypothesized that Kif4A could also induce THP-1-derived macrophages with suppressor cells’ features upon CCL2. As we expected, co-cultured with Cal-27 could significantly improved suppressive molecules of myelomonocytic cell-derived MDSCs, such as ARG-1, COX2, IDO1 and IL-4Rα in THP-1-derived macrophages (Fig. 5a). Whereas, treated THP-1-derived macrophages with siKif4A would reduce the increased level of ARG-1, COX2 and IL-4Rα in macrophages prominently (Fig. 5a). Similar results were observed when we treated Cal-27 cells in the upper chamber with siKif4A (Fig. 5b).

To further confirm whether Kif4A exerts the effect on promoting expression of candidate suppressive molecules of myelomonocytic cell-derived MDSCs via CCL2 signaling, CCL2 was neutralized at the beginning of co-culture. RTQ-PCR analysis showed that, expression of ARG-1, COX2 and IL-4Rα in THP-1-derived macrophages were significantly inhibited (Fig. 5c). We had proved that, CCL2 regulated expression of suppressive molecules of myelomonocytic cells via STAT3 signaling partially^10^. According to this result, we speculated that, Kif4A was an upstream signal that promoted STAT3 induction upon CCL2 in co-culture system. For this purpose, the effect of Kif4A on CCL2-STAT3 pathway was detected through western blot analysis. As showed in Fig. 5d, there was a statistically higher STAT3 protein expression in THP-1-derived macrophages from co-culture system as compared with monoculture group. This effect was abolished by treatment with a siKif4A on THP-1-derived macrophages (Fig. 5d).

Taken together, these findings support our hypothesis that Kif4A was an upstream signal that could induce THP-1-derived macrophages with myelomonocytic cell-derived MDSCs’ features upon CCL2.

## Discussion

In the current study, we utilized a two-chamber co-culture system to partly mimic the crosstalk between cancer cells and macrophages in OSCC tissue. The results provided experimental evidences that a remarkable interaction may occur between them, which gave rise to increasing expression of Kif4A in both two types of cells and elevated CCL2 level in supernatant. In addition, we identified the up-regulated expression of CCR2 on macrophages and high concentration of CCL2, which, in turn, promoted recruitment and polarization of macrophages, could be modulated by Kif4A.

Kinesin superfamily (Kif) constitute a group of microtubule-based motor proteins with 45 members that exhibit diverse functions, including the transport of vesicles, organelles, chromosomes, protein complexes, and so on^18, 19^. Several studies have identified that Kifs have intimate connection with the genesis and progression of many tumors^12^. Among them, Kif4A is significantly up-regulated in OSCC, cervical and lung cancers, whereas it is down-regulated in gastric cancers^14–16, 20^. In line with above research *in vivo*, we find a high level expression of Kif4A in OSCC cell line from current study (Fig. 1a). Many reports suggested that overexpression of Kif4A is closely related to tumor size and has positive correlation with cancer proliferation and carcinogenesis^15, 16^. To the contrary, some study has reported that loss of Kif4A leads to tumor formation and acts as a primary trigger of tumorigenesis and overexpression of it inhibits proliferation of carcinoma cells *in vitro* and *in vivo* ^21, 22^. Owing to these conflicting results, more efforts are called for to better understand the complex roles Kif4A plays in different cancers development and progression.

Growing body of evidences indicates that the crosstalk between tumor cells and infiltrated macrophages determines the immune status of tumor microenvironment, which is closely correlated with tumor development and progression and clinical outcomes of OSCC patients^23, 24^. However, the interaction between tumor cells and infiltrated macrophages and the induction of macrophages towards M2 in OSCC has not been elucidated clearly. The current two-chamber transwell co-culture study shows there is a remarkable increased Kif4A expression in macrophages in response to OSCC cells (Fig. 1b). Recently, an association has been demonstrated between Kif4A and T-cell activation in the idiopathic inflammatory myopathies^13^. Notably, our unpublished results also find targeting Kif4A with siRNA in RAW264.7, a mouse macrophage cell line, might affect its phenotype and cytokines profile. Numerous studies indicate that polarization of immunocyte, including T-cell and macrophage, play an irreplaceable role in the establishment of tumor environment and usually associate with tumor outcome^25, 26^. It is well accepted that, during polarization of macrophage, there are significant changes in the cellular structure involving reorientation of cytoskeleton and microtubule-organizing centers, which mediate by motor proteins. All these considerations indicate that, Kifs are most likely to participate in the process of macrophages polarization especially during the interaction with cancer cells. The findings from present study indicate that Kif4A play a positive role in shifting macrophage polarization towards M2 polarized cells in OSCC microenvironment. To our knowledge, it is the first study demonstrating that Kif4A is involved in the operation of macrophage polarization.

Amounts of studies have proved CCL2 expression in numerous kinds of tumor including prostate, breast, lung, melanoma, ovary and multiple myeloma^8, 27, 28^. In agreement with these findings from the tumor microenvironment, crosstalk between macrophages and OSCC cells may accelerate the production of CCL2 in co-culture system (Fig. 2a). More importantly, we observe that, expression of CCL2 in both two types’ cells is significantly inhibited upon Kif4A silencing via siRNA, suggesting a potential modulatory-supportive role of this motor protein in establishment of tumor microenvironment in OSCC. Being an important couple of chemokines, CCL2 mediate macrophages migration via binding CCR2. Several cancer cell lines, which express CCR2, shift their proliferation and invasion capability in response to CCL2, indicating the presence of CCR2 as a functional receptor^29, 30^. In the data presented here, an elevated CCR2 level in macrophages from co-culture system is found and associate with Kif4A partly. Our current study verifies a previously unrecognized novel role of Kif4A in positively regulating CCL2 expression and promoting macrophages migration toward OSCC cells. Moreover, no CCR2 can be detected in OSCC cell line, suggesting CCL2 might not operate OSCC progression via CCL2-CCR2 axis directly.

Recent exertion has brought new light on education of immunocyte, which connects CCL2 with the establishment of tolerant microenvironment^10, 11, 17, 28^. Besides the dramatic chemotaxis of CCL2, it has been proved to control T cells polarize towards T_H_2 cells with an up-regulated secretion of T_H_2 cytokine^11^. Furthermore, our previous study also demonstrates that, CCL2 plays a substantial role in the induction CD14^+^ myeloid-derived suppressor cells (MDSCs), and promotes a tolerogenic immune response^10^. CD14^+^ MDSCs with powerful immunosuppressive activity are first found in squamous cell carcinoma of the head and neck^31^. Both CD14^+^ MDSCs and tumor infiltrated macrophage differentiate from CD14^+^ myelomonocytic cells, with similar function in tumor environment. Our results prove that, Kif4A-CCL2 axis is involved in the promotion of macrophage polarization towards M2 (Fig. 4c) and up-regulation of several key suppressive molecules, including ARG1, COX2, and IL4Rα (Fig. 5), during interaction between macrophages and OSCC cells. These results might give an explanation why overexpressed Kif4A in OSCC correlated with poor clinical outcome.

Taken together, our current research provide substantial clues to better understand the mechanism that might involved in the induction and maintenance of tolerant microenvironment in OSCC. We identify the Kif4A-CCL2/CCR2-macrophage axis as a feasible new target to improve the clinical outcome of OSCC. However, the precise regulatory mechanisms of Kif4A-CCL2/CCR2-macrophage axis in this context have not been elucidated completely. Further investigations are still called for to obtain the panorama of the immunologic functions of Kif4A-CCL2/CCR2-macrophage axis in OSCC tissue.

## Materials and Methods

### Reagents and antibodies

For western blot analysis, the rabbit polyclonal to Kif4A was obtained from Abacm (Cambridge, UK), mouse anti-human CCR2 mAb was from Novus Biologicals (Littleton, CO, USA), rabbit anti-human mAb STAT3, anti-human mAb phosphor-STAT3 were from Cell Signaling Technology (Boston, MA, USA), mouse anti-human β-actin mAb and mouse anti-human GADPH mAb were purchased from Cell Signaling Technology (Beverly, MA, USA). The fluorescently conjugated anti-human mAbs CCR2-PE and the isotype antibody for flow cytometry analysis were obtained from Becton-Dickinson Biosciences (San Diego, CA, USA). Mouse anti-human CCL2 neutralizing antibody and isotype antibodies were purchased from R&D Systems (Minneapolis, MN, USA).

### Cell culture and generation of THP-1-derived macrophages

Cal-27 (human OSCC cell line) and THP-1 (human acute monocytic leukemia cell line) were obtained from the American Type Culture Collection (ATCC). Cells were maintained in complete RPMI-1640 medium (Gibco-Invitrogen, Carlsbad, CA, USA) with 10% fetal bovine serum (FBS, Gibco-Invitrogen, Carlsbad, CA, USA), 100 U/mL penicillin and 100 μg/mL streptomycin. All cells were cultured at 37 °C in a humidified 5% CO_2_ and 95% air environment. To generate THP-1-derived macrophages, 1 × 10^6^ THP-1 cells were treated with 100 ng/mL PMA for 48 h^32^ and adherent cells were collected for further research.

### Co-culture system studies

Cal-27 cells were seeded (2 mL/well, 1 × 10^5^/mL) into wells of a 6-well cell tissue culture plate (BD Biosciences; Franklin Lakes, NJ, USA) in RPMI-1640 medium with 10% FBS and allowed to attach overnight. Then medium was replaced by 2 mL pre-warmed fresh RPMI-1640 medium with 10% FBS, and THP-1-derived macrophages (1 mL/well, 2 × 10^5^/mL) were seeded into the upper 0.4 μm transwell chamber (BD Biosciences; Franklin Lakes, NJ, USA). Cal-27 cells and THP-1-derived macrophages were cultured alone in 6-well plate (2 mL, 2 × 10^5^/mL) fresh medium as monocultures control groups. In some co-culture experiments, as shown in Fig. 1c and d, THP-1-derived macrophages were seeded at different density or co-cultured for different time in 2 × 105/mL. After the co-culture for 24 h or other specific period of time, the co-culture cell-free supernatants and two chambers of cells were collected respectively. In order to detect the level of CCL2 in Cal-27 cells and THP-1-derived macrophages or in the supernatants of co-culture system, as shown in Fig. 2a and b, Cal-27 cells and THP-1-derived macrophages were co-cultured for 24 h, thereafter separated and cultured alone in 24-well plate with 1 mL pre-warmed fresh medium for another 24 h, then cells and cell-free supernatants were collected respectively. In order to detect CCL2 function in modulate the cytokines expression of THP-1-derived macrophages in co-culture system, as shown in Figs 4c and 5c, the co-culture system added 10 μg/mL anti-CCL2 neutralizing mAb for 24 hours, and then the cell-free supernatants and cells were obtained.

### shRNA transient transfection

The pGPU6/GFP/Neo vector containing Kif4A-targeting shRNA (siKif4A) sequences and a nonspecific shRNA (scr) as negative control were purchased from Shanghai GeneChem Corporation (Shanghai, China). THP-1-derived macrophages and Cal-27 cells were seeded in 6-well culture plates with 60% confluence before transfection. Cells then were transfected with siKif4A or scr (2 µg plasmid) by 5 µL Lipofectamine™ 2000 (Invitrogen, Carlsbad, USA) following the manufacturer’s instructions. Flow cytometry analysis showed that transfection efficiency at 48 h was more than 90%.

### Cell migration assay

Cell migration assay was carried out using 24-well transwell inserts (8 μm pore size membrane, BD Biosciences, Franklin Lakes, NJ, USA) following to the manufacturer’s instructions. In brief, THP-1-derived macrophages (1 × 10^5^ cells/well) or Cal-27 cells (1 × 10^5^ cells/well) were placed into the upper chamber of transwell inserts with 100 μL of serum-free medium. THP-1-derived macrophages (1 × 10^5^ cells/well, for migration assay of macrophages) or Cal-27 cells (1 × 10^5^ cells/well, for migration assay of cancer cells) in 600 μL RPMI 1640 medium containing 10% FBS were added into the lower chambers of the transwell plate correspondingly, 600 μL RPMI 1640 medium containing 10% FBS in lower chambers without cells as medium control. In some cases, CCL2 neutralizing antibody and isotype antibodies (10 μg/mL) were added into supernatant in lower chamber before incubation. Cells were incubated at 37 °C in 5% CO_2_ for 24 h. The migrated cells on the lower side of the membrane were fixed by 10% formalin and stained with eosin. Five random fields of each well were photographed and counted.

### Flow cytometric analysis

To detect the expression of CCR2 in THP-1-derived macrophages from co-culture system, cells were stained with fluorescence-conjugated anti-human mAbs CCR2-PE and the isotype antibody respectively for 30 minutes in the dark at room temperature. Cells acquisition was performed using a FACS Calibur flow cytometer (BD Biosciences, CA, USA) and data were analyzed via CellQuest software program of the FACS Calibur system (BD Biosciences, CA, USA).

### Chemokine analysis

The supernates were obtained from different culture groups and centrifuged at 2000 g, then stored in liquid nitrogen immediately until use. The amount of human chemokine, including CCL2, CCL7 in the co-culture system and monoculture groups supernatants were measured by enzyme-linked immunosorbent assay (ELISA) kit (R&D Systems, UK) according to the manufacturer’s guidelines. All measurements were performed in triplicate to avoid technical error and intra-assay variants

### Real-time quantitative RT-PCR (RTQ-PCR)

Total RNA of different culture groups was isolated with the Qiagen RNeasy mini kit (Hilden, Germany) according to the manufacturer’s instructions. RNA was reverse-transcribed to cDNA by RevertAid M-MuLV Reverse Transcriptase (Fermentas, Burlington, Ontario, Canada) with Oligo dT primers (Invitrogen, Carlsbad, CA, USA). Amplification of targeting gene were carried out by RTQ-PCR using the SYBR Green I (Bio-Rad, München, Germany) method in 10 μL reactions (5 μL of × 2 TaqMan Master mix, 1 μL each primer, 1 μL cDNA, 2 μL H2O). All procedures were conducted according to the manufacturer’s instructions. RTQ-PCR was performed using the LightCycler 2.0 Instrument (Roche, Penzberg, Germany). Relative gene expression was normalized to the expression of GAPDH mRNA and expressed as fold change for each relative gene reaction among the samples.

### Western blotting

Cell lysates containing equal amounts of total proteins from each group cells was separated on an 8% sodium dodecyl sulfate-polyacrylamide gel for Kif4A, CCR2, STAT-3, pSTAT-3, β-actin and GAPDH, respectively. The proteins were transferred to polyvinyl difluoride membranes (Millipore, USA), which were blocked with TBST (Tris-buffered saline, 0.1% Tween 20) containing 5% nonfat dried milk and immunoblotted with appropriate primary antibodies overnight at 4 °C. Membranes were washed with TBST for 3 times; the bounded antibodies were visualized using peroxidase-conjugated secondary antibodies (Santa Cruz Biotechnology, CA, USA) for 1 h. Results were visualized by chemiluminescence detection using an ECL Kit (Millipore, USA) on Image Station 4000 MM Pro (Carestream Health Inc., USA).

### Immunohistochemistry (IHC)

4-μm-thick sequential histological tumor sections were obtained from a representative formalin-fixed, paraffin-embedded OSCC tumor block and used for IHC analysis. IHC was performed using an automated staining system with antibodies against Kif4A (GTX115759, dilution 1:200; Gene Tex, USA), CD68 (lone KP1, dilution 1:100; ZM-006, ZSGB-bio, Beijng, China). Expression of all of the markers in cells was detected using a Novocastra Bond Polymer Refine Detection kit (PV9000, ZSGB-bio, Beijng, China) with a diaminobenzidinereaction to detect antibody labeling and hematoxylin counterstaining. Samples were viewed under the Olympus IX81 microscope.

### Statistical analysis

Prism 5.0 (GraphPad Software, CA, USA) was used for Statistical analysis. First, we used Shapiro-Wilk test to analysis normality of the data. If data were normally distributed, we used the 2-tailed Student’s t-test and Pearson’s test. Non-normally distributed data were analyzed by Mann-Whitney U-test. For all statistical tests, p values < 0.05 were considered statistically significant. Data from independent experiments were presented as the mean values ± standard error of the mean (mean ± SEM) for percentages.

## Electronic supplementary material

Supplementary Figure 1
