# Supplementary material for: Kif4A mediate the accumulation and reeducation of THP-1 derived macrophages via regulation of CCL2-CCR2 expression in crosstalking with OSCC
Source: Sci Rep. 2017 May 22;7:2226. doi: 10.1038/s41598-017-02261-7 (PMC5440405; doi:10.1038/s41598-017-02261-7)
Supplement: Supplementary file 1 — Supplementary Figure 1 [file 41598_2017_2261_MOESM1_ESM.pdf]

Kif4A mediate the accumulation and reeducation of THP-1 derived macrophages via regulation of CCL2-CCR2 expression in crosstalking with OSCC

Yun Zhang[1, 3], Shaohua Liu[1], Daiwei Qu[2], Ketao Wang[1], Lin Zhang[3], Xuanxuan Jing[3], Chen Li[3], Fengcai Wei[1], Xun Qu[3, *]

[1] Institute of Stomatology, Qilu Hospital of Shandong University, Jinan, 250012, Shandong, P.R. China

[2] Department of Gynaecology and Obstetrics, Qilu Hospital of Shandong University, Jinan, 250012, Shandong, P.R. China

[3] Institute of Basic Medical Sciences, Qilu Hospital of Shandong University, Jinan, 250012, Shandong, P.R. China

[*]Correspondence and requests for materials should be addressed to X.Q. (email: quxun@sdu.edu.cn)

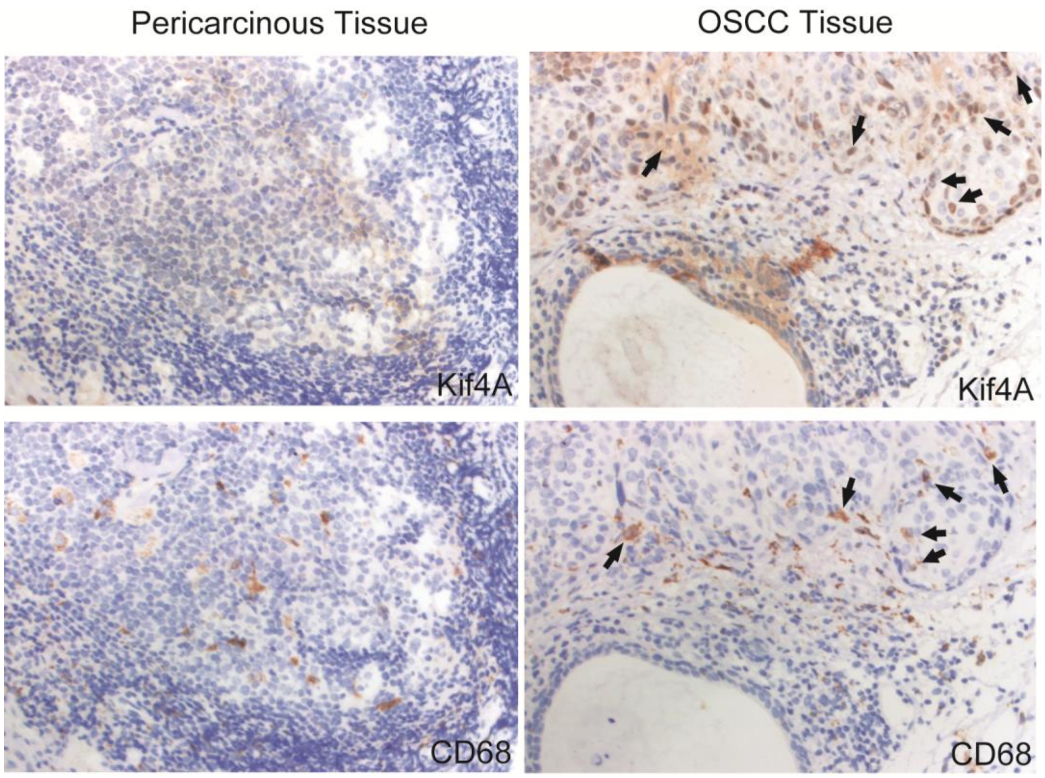

Supplementary Figure S1. Immunohistochemical analysis of CD68 and Kif4A expression in the OSCC tissue. 4-μm-thick sequential histological tumor sections were obtained from a representative formalin-fixed, paraffin-embedded OSCC tumor block and used for IHC analysis. After staining experiments with anti-CD68 mAb and anti-Kif4A mAb, double-positive cells (CD68$^+$ macrophages which express Kif4A) were shown in black arrowheads.
